# UBA2A regulates seed dormancy and the stability of chromatin‐retained *DOG1* messenger RNA

**DOI:** 10.1111/jipb.70056

**Published:** 2025-10-23

**Authors:** Ce Wang, Lien Brzeźniak, Sebastian Sacharowski, Michal Krzyszton, Veena Halale Manjunath, Mateusz Jan Olechowski, Anna Kulik, Szymon Swiezewski

**Affiliations:** ^1^ Laboratory of Seeds Molecular Biology, Institute of Biochemistry and Biophysics, Polish Academy of Sciences Warsaw 02‐106 Poland; ^2^ Doctoral School of Molecular Biology and Biological Chemistry, Institute of Biochemistry and Biophysics of the Polish Academy of Sciences Warsaw 02‐106 Poland; ^3^ Department of Biology University of Fribourg Fribourg 1700 Switzerland; ^4^ Laboratory of Plant Protein Phosphorylation, Institute of Biochemistry and Biophysics, Polish Academy of Sciences Warsaw 02‐106 Poland

**Keywords:** DOG1 regulation, post‐transcriptional regulation, seed dormancy

## Abstract

Multiple factors control primary seed dormancy established during seed maturation and secondary seed dormancy initiated when a non‐dormant imbibed seed is exposed to adverse conditions. A key player in the control of primary and secondary dormancy in *Arabidopsis thaliana* is the *Delay of Germination 1* (*DOG1*) gene, the expression of which is extensively regulated at the transcriptional and co‐transcriptional levels. Despite its importance, the influence of post‐transcriptional messenger RNA (mRNA) processing and mRNA storage of *DOG1* on the determination of dormancy depth remains elusive. Here, we show that the UBA2A protein, a member of the heterogeneous nuclear ribonucleoprotein (hnRNP) family, negatively regulates primary and secondary seed dormancy through the regulation of the *DOG1* gene expression at the post‐transcriptional level. *uba2a* mutants show higher levels of the *DOG1* mRNA. Surprisingly, *DOG1* gene transcription is not affected, as demonstrated by single‐molecule fluorescent *in situ* hybridization, chromatin‐attached mRNA analysis and Pol II chromatin immunoprecipitation (ChIP). Instead, our results show that the UBA2A protein decreases the stability of both chromatin‐bound and cytoplasmic *DOG1* mRNA pools, and results in higher chromatin retention of *DOG1* mRNA in the *uba2a* mutant. Our study highlights chromatin retention and mRNA stability as important features of *DOG1* gene expression regulation with a profound impact on dormancy establishment and shows that UBA2A protein, like its human homolog hnRNPAB, is most likely implicated in mRNA transport in the cell.

## Graphical abstract

In *Arabidopsis*, expression of *DELAY OF GERMINATION 1 (DOG1)*, a key regulator of seed dormancy, is regulated not only at the level of transcription but also post‐transcriptionally, at the level of mRNA stability.

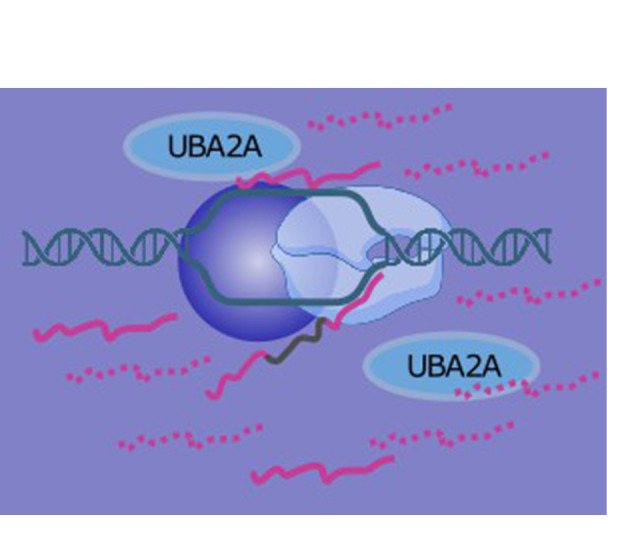

## INTRODUCTION

Seed dormancy is one of many fascinating seed adaptations that allow plants to traverse space and time. It is defined as the inability of a viable seed to germinate despite favorable conditions (Bentsink and Koornneef, 2008). Dormancy levels are intrinsically linked with germination speed, and low dormancy of seed pools has been reported to result in faster and more uniform seedling establishment (Soppe and Bentsink, 2020). Arabidopsis seeds acquire dormancy during seed maturation. This primary dormancy can be released by dry storage (known as after‐ripening) or cold treatment of imbibed seeds (stratification) (Bentsink and Koornneef, 2008). The Arabidopsis Col‐0 accession has a relatively low level of primary dormancy (Bentsink and Koornneef, 2008). This, along with the asynchronous seed production in Arabidopsis, makes primary dormancy challenging to study. Also, developmental defects in mutants, including changes in plant morphology or flowering time, can be easily mistaken for primary dormancy defects as they result in changes in seed maturation time between mutant and wild type (WT).

In many plant species, non‐dormant seeds can be induced into secondary dormancy when exposed to unfavorable conditions during imbibition (Lamont and Pausas, 2023). We and others have shown that imbibed Arabidopsis seeds that lost dormancy can be induced into secondary dormancy by heat treatment in the dark (Ibarra et al., 2016; Buijs, 2020). Secondary dormancy establishment‐release cycles are very important as they form the basis of the dormancy cycling phenomenon, which allows seed pools to survive and germinate for extended periods of time in soil seed banks (Footitt et al., 2011). Levels of secondary dormancy induction have been reported to depend on primary dormancy strength (Auge et al., 2015). Few genetic factors affecting secondary dormancy have been reported. This includes a quadruple nine‐cis‐epoxycarotenoid dioxygenase 2/5/6/9 mutant (*nced2/5/6/9*), a factor defective in abscisic acid (ABA) production. Also, mutants in several phytochrome genes, including *PHYD* (Martel et al., 2018) and *PHYB* (Arana et al., 2017), are defective in secondary dormancy induction (Bentsink and Koornneef, 2008). Importantly, secondary dormancy induced in after‐ripened seeds is less affected by developmental changes during seed maturation, which easily perturbs primary dormancy analysis. Despite this, secondary dormancy is less often used to study dormancy‐related phenomena (Angelovici et al., 2010). One of the key regulators of seed dormancy is the *DELAY OF GERMINATION 1* (*DOG1*) gene. *DOG1* was first identified as a quantitative trait locus (QTL) for dormancy variation among different accessions and was later confirmed to have a strong genome‐wide association (GWA study) with the dormancy level (Bentsink et al., 2006; Vidigal et al., 2016; Née et al., 2017; Nishimura et al., 2018). *DOG1* loss‐of‐function mutants, including *dog1‐3*, which has a T‐DNA insertion into the *DOG1* proximal promoter, have lower *DOG1* expression and weak primary and secondary dormancy (Huo et al., 2016). The function of the DOG1 protein is not fully understood, but it has been shown to bind and inhibit several PP2C phosphatases (Née et al., 2017; Nishimura et al., 2018).

*DOG1* transcription initiation is controlled by multiple transcription factors, including BZIP67, activating *DOG1* during seed maturation in response to cold, ERF12 that represses *DOG1* expression in the absence of ethylene and VAL1/VAL2 B3 domain‐containing transcription factors that repress *DOG1* expression by recruiting Polycomb Responsive Complex 2 (Bryant et al., 2019; Li et al., 2019; Chen et al., 2020). Factors involved in co‐transcriptional *DOG1* gene expression regulation include TFIIS, a Pol II complex auxiliary factor and AtNTR1 spliceosome accessory factor that both modulate *DOG1* gene transcription and alternative splicing (Grasser et al., 2009; Dolata et al., 2015). FY and CSTF77 polyadenylation and messenger RNA (mRNA) 3′ end formation proteins, which are other co‐transcriptional factors, affect *DOG1* mRNA 3′ end formation (Cyrek et al., 2016). The *DOG1* gene produces two alternative polyadenylation isoforms (APA): a short two‐exon proximally polyadenylated mRNA (*shDOG1*) and a long three‐exon distally polyadenylated mRNA (*lgDOG1*) (Nakabayashi et al., 2015; Cyrek et al., 2016). *shDOG1* is the predominant isoform in terms of mRNA level in seeds, and it can rescue the *dog1* mutant phenotype when expressed from the endogenous promoter, suggesting that it is the most functional *DOG1* isoform (Bryant et al., 2019; Chen et al., 2020; Cyrek et al., 2016; Li et al., 2019). Finally, *DOG1* expression is controlled by at least three long non‐coding RNAs (lncRNAs): *1GOD*, *PUPPIES*, and *MUSHER* (Fedak et al., 2016; Montez et al., 2023; Sacharowski et al., 2025). The mechanisms of action of these lncRNAs are not fully elucidated. The *1GOD* antisense originates near the *DOG1* proximal APA site and decreases *DOG1* expression. This is supported by the analysis of a *dog1‐5* mutant that has a T‐DNA inserted in the *1GOD* promoter, resulting in low *1GOD* and high *DOG1* transcript levels (Fedak et al., 2016). The *PUPPIES* lncRNA, which originates from the *DOG1* promoter, enhances *DOG1* expression by augmenting Pol II pausing at *DOG1* exon 1 and enhancing *DOG1* transcriptional bursting, as revealed by single‐molecule fluorescent *in situ* hybridization (smFISH) (Montez et al., 2023). Another lncRNA that positively regulates *DOG1* expression is *MUSHER*. Transcribed from the region downstream of *DOG1*, *MUSHER* interacts with mRNA cleavage and polyadenylation factors and promotes *DOG1* proximal poly(A) site usage (Sacharowski et al., 2025). As described above, the *DOG1* gene expression is extensively regulated at the transcriptional and co‐transcriptional levels (Carrillo‐Barral et al., 2020). Nothing is known about *DOG1* expression control through mRNA stability, mRNA storage or export. This could be partially due to the technical limitations of working with seeds. Nevertheless, the mRNAs preserved in the mature seed are considered to be crucial for the start of germination without relying much on active transcription, necessary for later seedlings' growth (Marvin and Inada, 2014; Sajeev et al., 2019).

In 2002, Witold Filipowicz's laboratory identified Arabidopsis UBP1‐associated protein 2a (UBA2A) as an interactor of poly(A) binding protein UBP1 (Lambermon et al., 2002). They demonstrated that UBA2A, like UBP1, is a nuclear protein that can bind RNA, which suggested UBA2A's role in RNA metabolism (Lambermon et al., 2002; Riera et al., 2006). Arabidopsis UBA2A protein, as well as its close homolog in *Vicia faba* (AKIP1), was later found to be phosphorylated by ABA‐dependent kinase (Li et al., 2002; Riera et al., 2006). The *UBA2A* gene overexpression and knockout plants were reported to show no phenotypes, including the germination speed of non‐dormant seeds (Riera et al., 2006). UBA2A belongs to the heterogeneous nuclear ribonucleoprotein (hnRNP) protein family, with hnRNPAB being its closest human homolog. In agreement, in Arabidopsis, UBA2A has been shown to be localized in the nucleus (Riera et al., 2006). hnRNP is a diverse family of RNA‐binding proteins that participate in transcriptional and post‐transcriptional regulation of gene expression (Geuens et al., 2016). Not a lot is known about the *hnRNPAB* gene functions even in humans. Its expression is misregulated in multiple cancers (Wang et al., 2023), and the hnRNPAB protein was shown to bind influenza A virus RNA, enhancing its nuclear retention (Wang et al., 2021).

Here, using comprehensive phenotyping of both primary and secondary dormancy, as well as genetic interaction with the *dog1* mutant, we show that the UBA2A protein negatively regulates dormancy through the *DOG1* gene expression regulation. Using Pol II chromatin immunoprecipitation (ChIP), smFISH and chromatin fractionation, we show that UBA2A does not affect *DOG1* gene transcription. However, our results demonstrate that *uba2a* mutants have higher *DOG1* mRNA retention at chromatin and *DOG1* mRNA stability, leading to *DOG1 mRNA* and protein accumulation. This shows that the post‐transcriptional level of gene expression regulation is important for a full understanding of seed dormancy control in plants.

## RESULTS

### UBA2A negatively regulates seed dormancy

**Figure 1:**
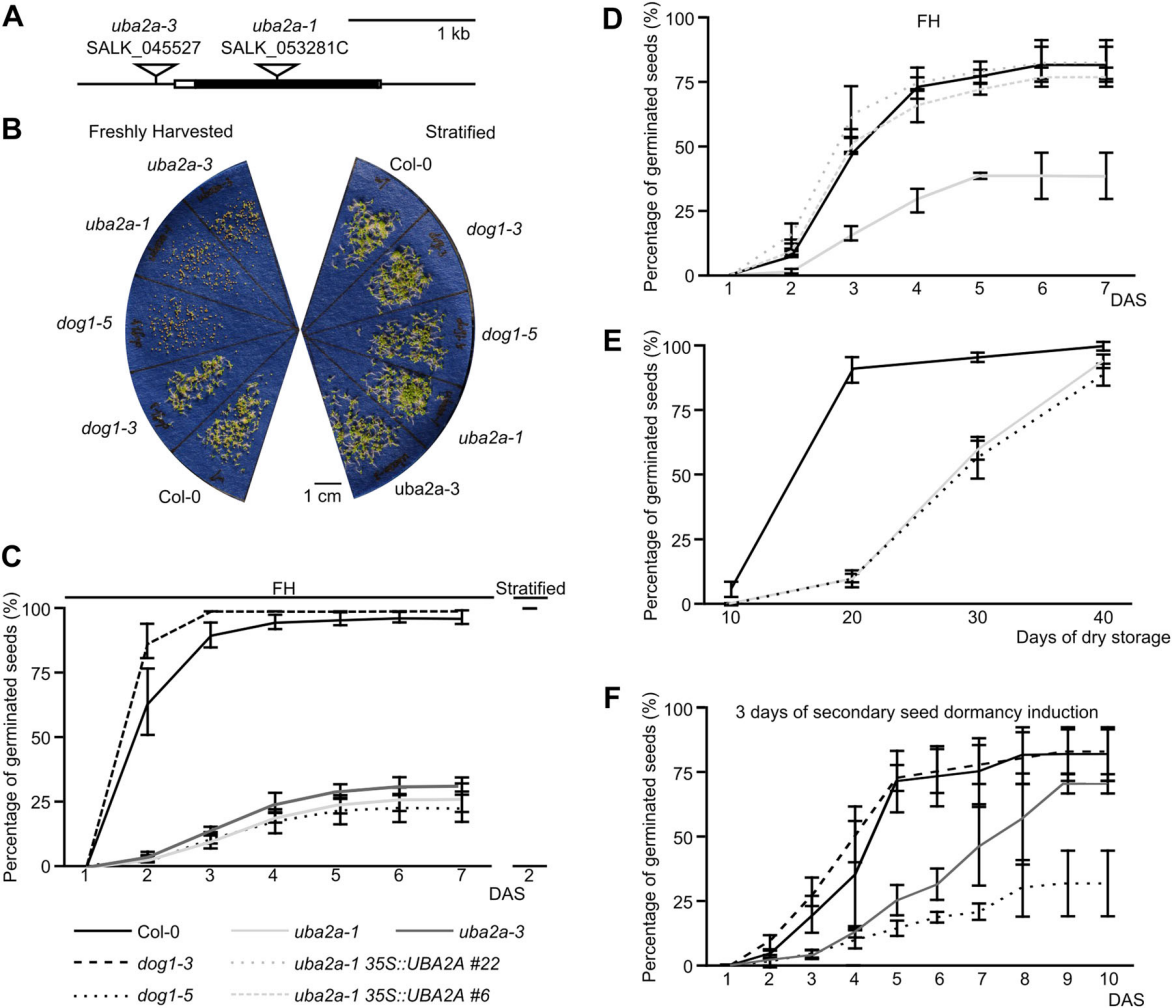
UBA2A negatively regulates primary and secondary seed dormancy *DOG1* gene mutants were used for comparison, with *dog1‐3* showing weaker and *dog1‐5* showing stronger dormancy. **(A)** *UBA2A* gene structure with positions of T‐DNA insertions in the mutants used in this work. A black rectangle corresponds to an exon, and white rectangles correspond to untranslated regions (UTRs). **(B)** An example picture for the primary dormancy test results. Dormancy test results for freshly harvested and stratified seeds. Pictures were taken 3 d after sowing (DAS). **(C)** Both tested *uba2a* mutant alleles show strong primary seed dormancy. Freshly harvested (FH) seeds were sown and scored for germination at 7 DAS. A stratification control is shown on the right side of the graph for the same lot of seeds. **(D)** Ectopic expression of *UBA2A* mRNA complements the *uba2a‐1* mutant primary dormancy defects. Freshly harvested mutant and two complementation line seeds were sown and scored for germination up to 7 DAS. **(E)** The *uba2a* mutant requires more than 30 d of after‐ripening for full dormancy release. Seeds were stored in dry conditions for the indicated time and scored for germination at 3 DAS. **(F)** UBA2A negatively regulates secondary dormancy induction. Fully after‐ripened seeds were induced into secondary dormancy for 3 d and scored for germination up to 10 DAS. All graphs show the mean germination percentage calculated from four biological experiments. Error bars denote standard deviations.

We noticed that freshly harvested seeds from two independent *UBA2A* gene T‐DNA insertion lines, *uba2a‐1* and *uba2a‐3*, had reduced germination (Figure 1A, B). Germination defect was reversed by stratification, as almost 100% of *uba2a* seeds established seedlings after transferring from cold treatment to permissive conditions (Figure 1B, C). This indicates that the *UBA2A* gene affects dormancy rather than seed viability. Both *uba2a* alleles exhibited a strong decrease in *UBA2A* mRNA expression, suggesting they are both null mutants of the *UBA2A* gene (Figure S1A). Importantly, two independent *35S*::*UBA2A* transgenic lines in the *uba2a*‐1 background expressed UBA2A and reversed the *uba2a*‐1 seed dormancy phenotype (Figures 1D, S1B). To additionally confirm that *uba2a* mutant has enhanced seed dormancy, we tested whether the germination defect of *uba2a* seeds could be alleviated by after‐ripening. Indeed, *uba2a‐1* seeds germinated at comparable rates to Col‐0 after prolonged dry storage. Importantly, *uba2a‐1* seeds behaved similarly to the *dog1‐5* mutant, which is characterized by strongly enhanced seed dormancy. Both mutants lost their dormancy only after 40 d of dry storage, while Col‐0 seeds reached maximum germination after < 20 d of storage (Figure 1E). Based on this, we conclude that UBA2A negatively regulates primary dormancy.

Subsequently, we used fully after‐ripened mutant and WT seeds for the secondary dormancy induction test, as previously described by us (Krzyszton et al., 2022). A partial induction of secondary dormancy and a delay of *uba2a‐1* germination compared to Col‐0 seeds after only 3 d of incubation were observed (Figure 1F). This shows that the *uba2a* mutants have enhanced primary seed dormancy levels and secondary seed dormancy induction when compared to Col‐0 plants.

UBA2B protein is a close sequence homolog of UBA2A in the Arabidopsis genome. We therefore obtained *UBA2B* gene T‐DNA lines *uba2b‐1* and *uba2b‐2* (Figure S1C). *uba2b‐2* mutant, which has a T‐DNA insertion at the beginning of the *UBA2B* gene protein coding sequence, showed no detectable expression of *UBA2B* mRNA (Figure S1D). Seed dormancy analysis revealed that *uba2b‐2* did not have obvious seed germination defects (Figure S1E). Consistently, the double mutant *uba2a‐3 uba2b‐2* displayed an enhanced dormancy phenotype that was very similar to the single *uba2a‐3* mutant (Figure S1E). Based on this, we reasoned that UBA2A controls dormancy independently from its homolog, UBA2B. Phenotypic analysis of *uba2a*, *uba2b* and *uba2a uba2b* double mutants at seedling, bolting and seed setting stages showed no marked developmental defects (Figure S2). This suggests that, under standard conditions, the defects in *UBA2A* gene expression are mainly manifested by changes in seed dormancy, and *UBA2A* regulation of dormancy is not redundant with *UBA2B*. In summary, our data demonstrate that *UBA2A* negatively regulates both primary and secondary dormancy in Arabidopsis (Figures 1, S1).

### *uba2a* shows high *DOG1* expression

**Figure 2:**
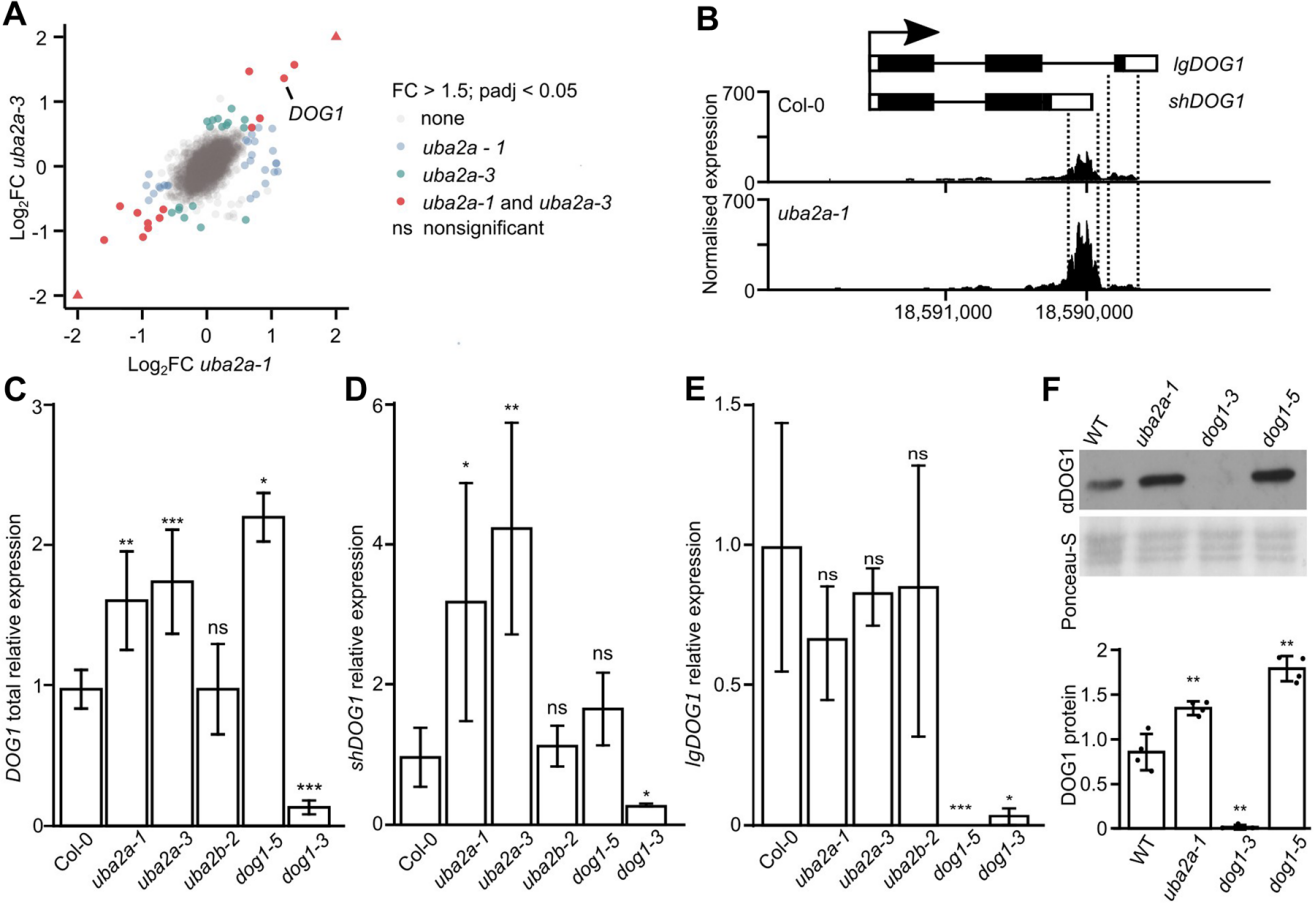
***uba2a*** **mutants show limited gene expression changes, including upregulation of short but not long** ***DOG1*** **alternative mRNA isoform** **(A)** 3′ RNA sequencing (RNA‐seq) in *uba2a‐1* and *uba2a‐3* shows only minor changes in gene expression. Dot plot for all genes Log_2_ fold change (FC) differences in both mutants compared to Col‐0. Genes with absolute FC > 1.5 and *P*_adj_ < 0.05 are color‐labeled if affected in *uba2a‐1*, *uba2a‐3* or both alleles. Triangles represent two outlier genes with absolute FC > 4. The experiment was performed in four biological replicates. **(B)** 3′ RNA‐seq reads for *DOG1* gene in Col‐0 and *uba2a‐1*. The upper panel shows the *lgDOG1* and *shDOG1* structures with exons shown as black rectangles, introns as lines and untranslated regions (UTRs) as white rectangles. The lower panel shows the normalized number of reads corresponding to positions along the *DOG1* gene, and the axis labeling shows chromosome 5 position. **(C–E)** Reverse transcription quantitative polymerase chain reaction (RT‐qPCR) was used to analyze the expression levels of **(C)** total *DOG1*, **(D)** short *DOG1* (*shDOG1*), and **(E)** long *DOG1* (*lgDOG1*) mRNA isoforms. Data represent the average from four biological replicates, normalized to Col‐0, and error bars denote *SD*. *DOG1* gene mutants were used for comparison. For comparison of Col‐0 and indicated mutants, *P*‐values were calculated using a Student's *t*‐test and denoted as follows: *P < 0.05, **P < 0.005, and ***P < 0.0001. *UBC21 (PEX4)* gene mRNA was used as a reference. **(F)** Western blot (upper panel) shows increased level of DOG1 protein in *uba2a‐1* seeds compared to Col‐0. Ponceau‐S staining (lower panel) was used for sample loading control. Quantification over four biological replicates is shown on the right panel, pairwise comparisons with wild type (WT) were tested using Student's *t*‐tests, with significance denoted as **P < 0.005.

To identify the molecular defect underlying the observed strong dormancy phenotype, we performed 3′ RNA sequencing (RNA‐seq) (Krzyszton et al., 2022) in dry seeds of both *uba2a‐1* and *uba2a‐3* mutants. Differential gene expression analysis revealed very limited transcriptional changes, with only six genes upregulated and nine downregulated (fold change (FC) > 1.5, adjusted *P*‐value < 0.05) consistently in both alleles (Figure 2A; Table S1). Among the upregulated genes, we detected *DOG1*, a key regulator of dormancy (Carrillo‐Barral et al., 2020). A close inspection of reads mapping to *DOG1* showed a strong increase of reads mapping to the proximally polyadenylated *shDOG1* and only minor changes in read number mapping near the distal poly(A) site (Figures 2B, S1G), suggesting a specific effect of the *UBA2A* gene on the short but not long *DOG1* transcript versions.

Consistently, reverse transcription quantitative polymerase chain reaction (RT‐qPCR) showed an upregulation of an amplicon targeting all *DOG1* (Figure 2C) or short (Figure 2D) but not long (Figure 2E) transcript isoforms in both *uba2a* mutant alleles. The specific effect of *UBA2A* on *short* but not *lgDOG1* has been independently verified on seeds from plants grown in different seasons, seeds stored for different amounts of time, as well as in *uba2a* seedlings. In agreement with the absence of seed dormancy defects in the *uba2b* mutant, we observed no significant changes in any of the *DOG1* transcript versions in *uba2b‐2* (Figure 2C–E) and no difference in *shDOG1* splicing (Figure S4C). The increased expression of the *shDOG1* transcript in *uba2a* mutants could account for their enhanced seed dormancy phenotype, as *shDOG1* has been proposed to be the most functional *DOG1* mRNA isoform and shown to rescue the *dog1* mutant phenotype (Cyrek et al., 2016). To directly confirm that the seed phenotype observed in *uba2a‐1* is a result of increased DOG1 activity, we performed a western blot analysis using DOG1‐specific antibody (Figures 2F, S4D). Similar to mRNA levels, DOG1 protein in *uba2a‐1* was increased around 1.5‐fold compared to Col‐0 seeds, strengthening the final conclusion that *UBA2A* affects seed dormancy by controlling *DOG1* expression.

### *UBA2A* requires *DOG1* for seed dormancy control

**Figure 3:**
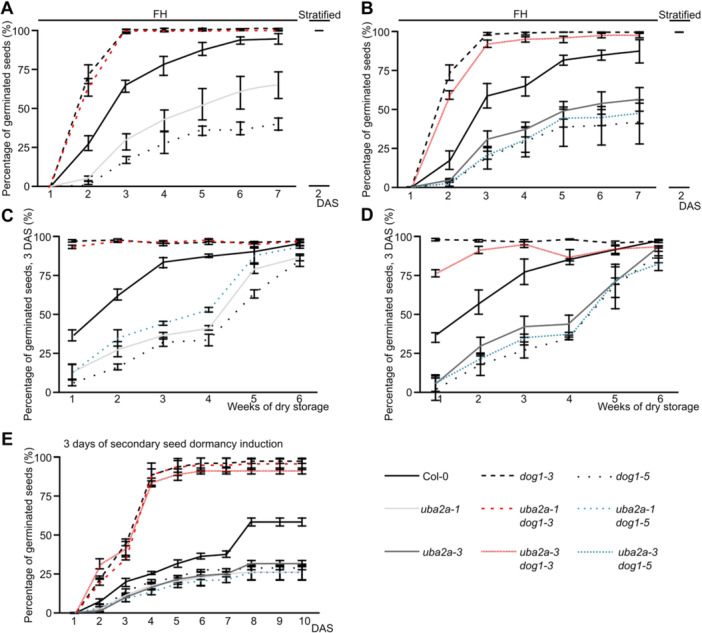
**UBA2A requires the functional** ***DOG1*** **gene for primary seed dormancy control** **(A**, **B)** Freshly harvested (FH) seeds were used to analyze the percentage of germinated seeds for 7 consecutive d after sowing (DAS) (left part) or after 2 d post‐stratification (right part) for **(A)** *uba2a‐1* and *uba2a‐1 dog1‐3* double mutant and **(B)** *uba2a‐3* and *uba2a‐3 dog1‐3*, *uba2a‐1 dog1‐5* double mutants. **(C**, **D)** Seeds were stored in dry conditions for the indicated time and scored for germination at 3 DAS for **(C)** *uba2a‐1* and *uba2a‐1 dog1‐3* double mutant and **(D)** *uba2a‐3* and *uba2a‐3 dog1‐3, uba2a‐1 dog1‐5* double mutants. **(E)** Fully after‐ripened seeds were induced into secondary dormancy for 3 d and scored for germination up to 10 DAS. All graphs show the mean germination percentage calculated from four biological experiments. Error bars denote *SD*s.

To examine whether the upregulation of *DOG1* gene expression is required for increased dormancy of *uba2a* mutants, we generated *uba2a‐1 dog1‐3* and *uba2a‐3 dog1‐3* double lines. Phenotypic analysis showed no major developmental defects at seedling, flowering or seed maturation stages in these lines (Figure S2). Germination analysis of freshly harvested seeds showed very rapid germination of both double and *dog1‐3* single mutants, with all seeds germinated within 3 d, in contrast to Col‐0, which required almost 7 d to reach full germination (Figure 3A, B). In contrast to Col‐0 and in agreement with our previous observations (Figure 1), *uba2a‐*1 and *uba2a‐3* single mutants had around 50% of seeds that did not germinate after 7 d, similar to the *dog1‐5* mutant. Our results show that the *DOG1* gene mutation suppressed the *uba2a* mutant seed dormancy phenotype, as both *uba2a‐1 dog1‐3* and *uba2a‐3 dog1‐3* lines had 100% germination at the end of the experiment. Similarly, all lines used in this experiment had 100% germination after stratification (Figure 3A, B) and after 6 weeks of dry storage (Figure 3C, D), confirming that *DOG1* mutation suppresses the *uba2a* primary seed dormancy phenotype (Figure S3, for seed germination at different d after sowing (DAS)).

### Accumulation of *DOG1* transcript at the transcription sites in *uba2a* seeds

**Figure 4:**
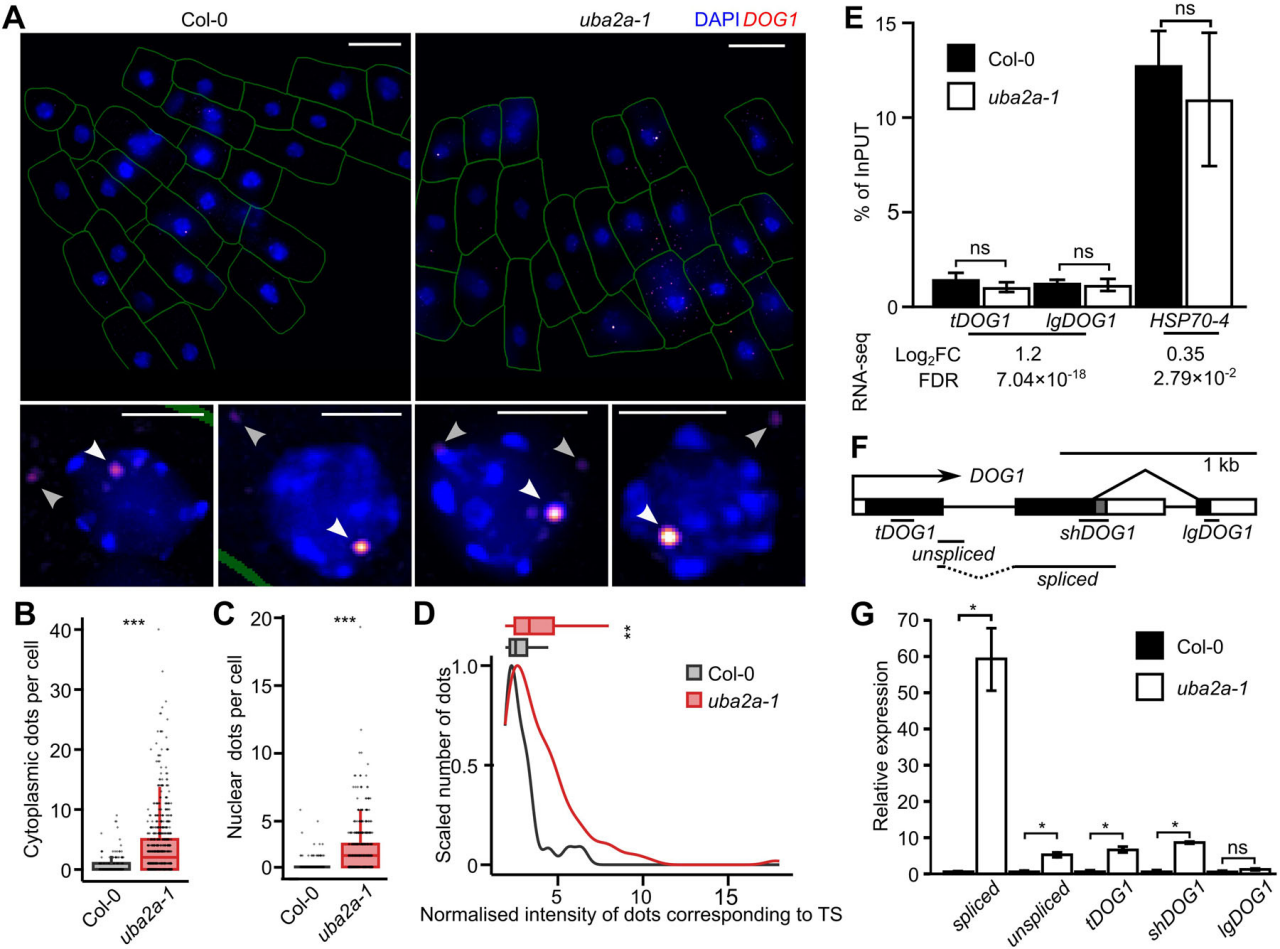
***uba2a*** **shows chromatin retention of** ***DOG1*** **mRNA** **(A)** Single‐molecule RNA fluorescence in *situ* hybridization (smFISH) imaging of *DOG1* RNA in seed cells. A representative picture of the radicle tip with two nuclei magnified below for Col‐0 and *uba2a‐1* mutant seeds (left and right panels, respectively). The cell wall is shown as a green line, 4ʹ,6‐diamidino‐2‐phenylindole (DAPI) staining in blue and *DOG1* signal in red‐to‐white scale. White arrows point to the transcription sites, and gray arrows point to cytoplasmic signals. Scale bars, 10 μm. **(B**, **C)** Quantification of dots corresponding to *DOG1* RNAs in **(B)** the cytoplasm and **(C)** the nucleus. **(D)** The scaled number of dots corresponding to the transcription sites (TS) plotted against the dot brightness normalized to the average cytoplasmic dot. TS dot brightness intensity is summarized above the density plot with boxplots (boxplot whiskers show a 1.5 interquartile range), **(B**, **C** and **D)**—statistical analysis was done using the Wilcoxon test: **P < 0.005 and ***P < 0.0001. **(E)** Pol II chromatin immunoprecipitation (ChIP) in dry seeds. Data represent percent of input normalized to Col‐0 (average from three biological replicates) and error bars denote *SD*s. Primers to the *ACT7* (*AT5G09810*) gene promoter were used as a reference. No differences were detected using a Student's *t*‐test. The lower panel displays the Log_2_ fold change in gene expression between the *uba2a‐1* mutant and Col‐0 and false discovery rate from 3′ RNA sequencing. **(F)** The *DOG1* gene structure with exons shown as black rectangles, introns as lines and untranslated regions (UTRs) as white rectangles. Amplicon positions for **(E)** and **(G)** are shown below the schematic, with the dotted line corresponding to the exon‐exon junction spanned by the forward primer. **(G)** Quantification of chromatin‐attached RNA by reverse transcription quantitative polymerase chain reaction (RT‐qPCR). Data represent the average from four biological replicates, normalized to Col‐0, and error bars denote *SD*. *ACT7* gene mRNA was used as a reference. The *P*‐values were calculated using a Student's *t*‐test, with significant differences to Col‐0 shown, *P < 0.05.

We used smFISH to analyze *DOG1* mRNA localization and transcription in WT and *uba2a‐1* dry seeds. The available probe set covers the entire sequence of the *shDOG1* transcript. Consistently with recent works (Montez et al., 2023; Fonseca et al., 2024), we detected two types of dots: low‐intensive present in both cytoplasm and nucleus that we assume correspond to single *DOG1* mRNA molecules (marked with the gray arrow; Figure 4A) and more intense dots localized in the nucleus (white arrow; Figure 4A) that we consider to correspond to transcription sites (TS; additional pictures shown in Figure S5). In accordance with the diploid nature of the Arabidopsis embryo, there are zero, one or two TS dots per nucleus. In the case of *DOG1* probes, we most often observed a single TS dot, which suggests that both alleles rarely initiate transcription at the same time, likely due to the low *DOG1* gene expression at final seed maturation stages (Bentsink et al., 2006; Fedak et al., 2016).

The quantification of dots corresponding to mRNA molecules showed that *uba2a‐1* has an increased number of *DOG1* transcripts per cell. Col‐0 plants had on average 1.77 cytoplasmic and 1.32 nuclear *DOG1* mRNA molecules, while the *uba2a‐1* mutant had a statistically significant increase with on average 4.93 cytoplasmic and 2.50 nuclear dots (Figure 4B, C). This result agrees with the FC of *DOG1* mRNA upregulation observed by RT‐qPCR (Figure 2C). We also observed a change in the intensity of the dot corresponding to TS (Figure 4A bottom panel and Figure 4D). In the *uba2a‐1* mutant, the nuclear dot corresponding to TS was on average 3.99 times more intense than a cytoplasmic dot, while it was only 2.91 times more intense in Col‐0. This may indicate a stronger association of *DOG1* mRNA with chromatin in the *uba2a* mutant.

We performed isolation of chromatin‐bound RNA fraction and used it to quantify the abundance of different versions of *DOG1* transcripts in WT and *uba2a1* mutant seeds (Montez et al., 2023). The nuclei fractionation was performed on dry seeds as well as on transcriptionally active seeds collected during maturation. Consistent with the effect of the *UBA2A* gene on short but not long *DOG1* transcript versions observed in total cell RNA (Figure 2), we detected an increase in total and short but not long *DOG1* mRNA in chromatin‐attached RNA fraction (Figures 4F, G, S4). In addition, we detected a similar increase in unspliced RNA molecules containing intron 1 (Figure 4G). Interestingly, we observed a massive, approximately 60‐fold increase in the amount of spliced full‐length *DOG1* transcripts bound at chromatin (Figure 4G), which suggests higher accumulation of mature mRNA than pre‐mRNA.

Next, we performed Pol II ChIP‐qPCR in dry seeds as well as in seeds collected during late maturation. Both showed no change of Pol II levels at *DOG1* chromatin in the *uba2a‐1* mutant compared to Col‐0 (Figures 4E, S4). We also assessed Pol II levels in *uba2a* on the *HSP70‐4* gene, which showed no change in expression in our 3′ RNA‐seq (Figures 4E, S4). Consistently, we observed no change in polymerase occupancy on *HSP70‐4*. This suggests that *UBA2A* does not regulate the *DOG1* gene at the transcriptional level. Based on unaffected Pol II levels at the *DOG1* gene, we attribute increased chromatin‐associated *DOG1* mRNA in the *uba2a* mutant to defective mRNA export from the transcription site.

### UBA2A regulates *DOG1* mRNA stability

**Figure 5:**
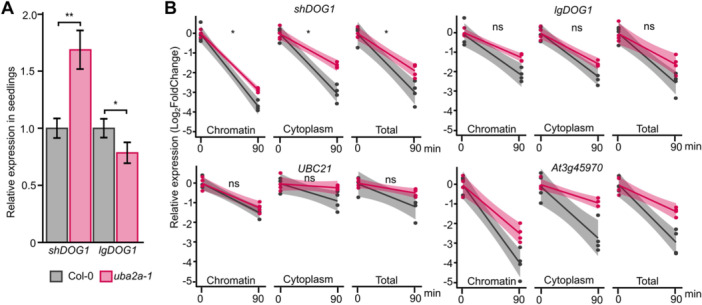
**The** ***uba2a*** **mutant shows increased stability of the** ***shDOG1*** **transcript** **(A)** Reverse transcription quantitative polymerase chain reaction (RT‐qPCR) quantification of *DOG1* gene transcript isoforms relative expression in 7 d after sowing (DAS) seedlings. Data were standardized to *UBC21* (*PEX4*) and represent the average from four biological replicates, normalized to Col‐0; error bars denote *SD*s. The *P*‐values were calculated using a Student's *t*‐test. **(B)** Flavopiridol mRNA stability assay in chromatin, cytoplasm and total cell fractions. Relative RNA level was measured in samples before (0 min) and after (90 min) flavopiridol spraying. The experiment was performed in four biological replicates. A linear regression model was fitted to Log_2_‐normalized data using standard curves. The coefficients were compared, and *P*‐values were calculated using the lsmeans R package. Significant differences in turnover rates (coefficients) between *uba2a* and Col‐0 are marked with *P < 0.05. The shaded area shows confidence intervals.

We next compared *DOG1* mRNA stability in *uba2a* and Col‐0 upon inhibition of Pol II transcription. To inhibit Pol II transcription, we used flavopiridol, which inhibits kinases involved in Pol II elongation activation (Chen et al., 2019). We could not perform mRNA stability directly in dry or developing seeds for a few reasons: (i) dry seeds are metabolically inactive; (ii) there are technical difficulties with compounds penetration through the seed coat; and (iii) *DOG1* mRNA levels show the high dynamics of changes during both seed maturation and imbibition which could blur the effects of transcription inhibition. Therefore, we used 7‐d‐old seedlings. First, we confirmed that the *DOG1* transcripts level changes in *uba2a‐1* seedlings are similar to those observed in dry seeds. Notably, the *shDOG1* transcript was increased in *uba2a‐1* seedlings compared to Col‐0, like in dry seeds, while the *lgDOG1* transcript was slightly decreased (Figure 5A).

## DISCUSSION

### UBA2A controls *DOG1* mRNA chromatin retention and mRNA stability

DOG1 protein is a major regulator of seed dormancy, and its expression is tightly linked to the dormancy level (Footitt et al., 2020). 3′ RNA‐seq in *uba2a* mutant seeds showed limited changes in gene expression with *DOG1* among the 15 misregulated genes. Among those genes, only *DOG1* mutants have been shown to affect seed dormancy, motivating us to explore its role in UBA2A‐mediated control of dormancy. The observed upregulation of *DOG1* transcript and protein levels in *uba2a* is consistent with the positive role of *DOG1* in dormancy control. Genetic analysis indicated that *DOG1* is indispensable for stronger dormancy observed in *uba2a*, as the *dog1* mutation can suppress the primary and secondary dormancy phenotypes of both tested *uba2a* alleles. We conclude that UBA2A controls dormancy through the *DOG1* gene.

Our data raises the question of why only a limited number of genes show a strong increase in expression in the *uba2a* mutant. Albeit unlikely, it is possible that UBA2A and speckles containing it may be important for *shDOG1* mRNA maturation. However, in our recent manuscript (Peter et al., 2024), we observed that many seed‐stored mRNAs show a high level of uridylation of their poly(A) tails, and among them, *DOG1* mRNA is one of the most strongly uridylated (Table S1). Uridylation of mRNAs is a post‐transcriptional modification postulated to help stabilize mRNAs (Sement et al., 2013), and mutants defective in uridylation show decreased levels of *DOG1* mRNA and seed dormancy (Peter et al., 2024). Importantly, UBA2A was originally described as an oligo(U) binding protein partner (Lambermon et al., 2002). However, uridylation cannot ensure specificity for UBA2A, as there are a few hundred uridylated mRNAs in mature dry seeds (Peter et al., 2024). Another property of *DOG1* mRNA is that it is very strongly downregulated during the final stage of seed maturation and desiccation (Bentsink et al., 2006; Fedak et al., 2016). We checked the recently published dataset of transcriptomic changes during Arabidopsis seed maturation and noticed that four out of five *uba2a* upregulated mRNAs show a strong drop in levels during late maturation, and three of them are strongly uridylated (Table S1) (Artur et al., 2024). Although probably those are not the only factors contributing to the selective effect of the *uba2a* mutation, they may be the most important.

**Figure 6:**
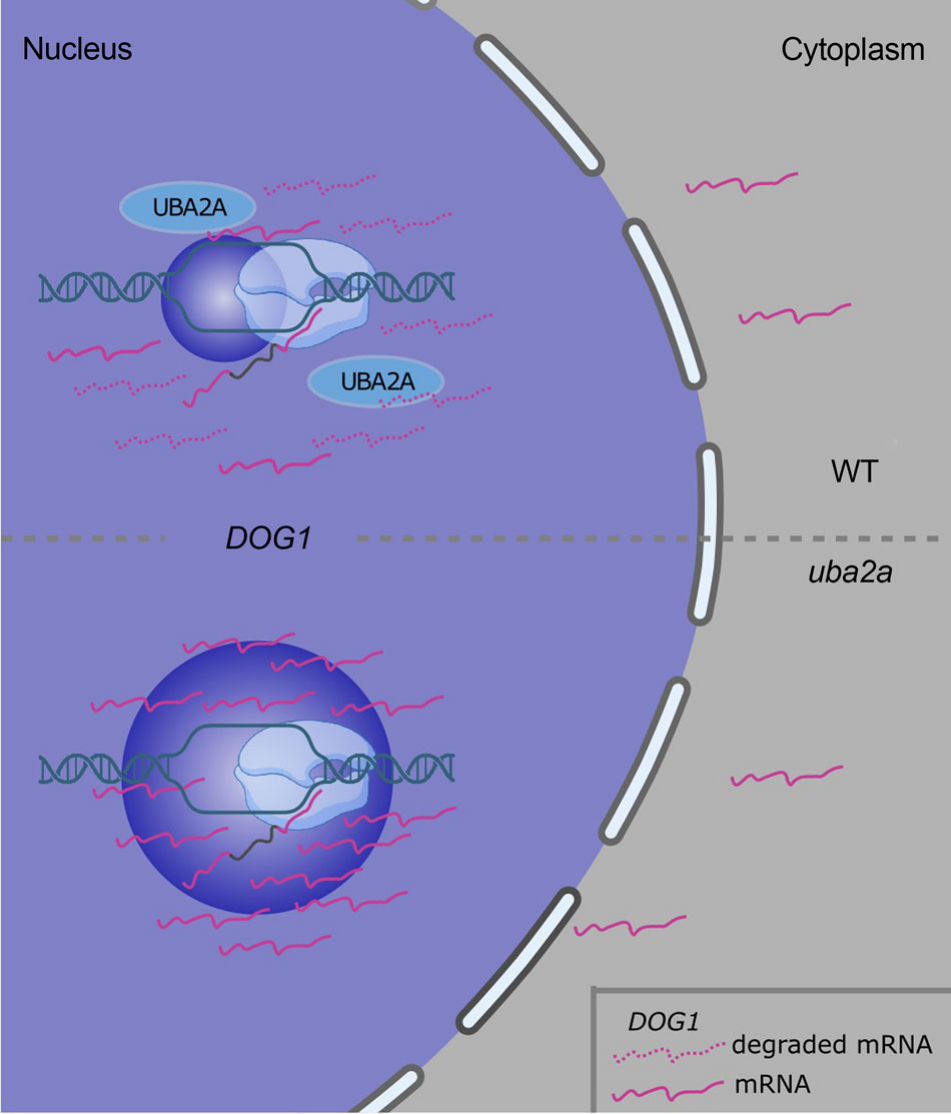
**Model for UBA2A involvement in** ***DOG1*** **mRNA chromatin retention. For details, see the discussion** The *uba2a* mutant shows chromatin retention of spliced *DOG1* mRNA at the site of transcription.

We used smFISH to analyze *DOG1* mRNA localization and observed an increased intensity of the dots considered TSs in the *uba2a* mutant which is in agreement with its higher *DOG1* mRNA association with chromatin, tested using cellular fractionation (Figure 4). A commonly made assumption is that an increase in TS dot intensity indicates an enhancement of transcription burst. Here, this is unlikely, since we observed no changes in Pol II occupation in *uba2a* (Figures 4, S4), suggesting a defect in mRNA export. In parallel, we performed the analysis of *DOG1* mRNA stability in cellular fractions, observing higher stability of *shDOG1* mRNA in the *uba2a* mutant in both chromatin and cytoplasm fractions (Figures 4, S5). However, it is still not clear if the increased *DOG1* mRNA retention at chromatin is linked to the observed increase in *DOG1* mRNA stability and level. One possible explanation could be that UBA2A is directly involved in mRNA export or some mRNA quality control process which takes place exclusively at the site of the transcription (Rambout and Maquat, 2024). This mechanism may be needed to remove superfluous or partially aberrant mRNA molecules. For example, in yeast, defects in factors related to pre‐mRNA 3′ processing result in the retention of mRNAs near TS (Paul and Montpetit, 2016). This is in line with the observed increase of *DOG1* mRNA stability and retention in the *uba2a* chromatin fraction (Figure 6). These mRNAs, temporarily stored at the site of transcription, would finally find their way to the cytoplasm, which is why we also observe the increase of cytoplasmic smFISH signal and global increase of mRNA levels in *uba2a* mutant.

Another explanation for increased stability and levels of *DOG1* mRNA could be an increase in chromatin retention itself, as several works proposed that nuclear‐stored mRNAs are more stable, which helps to buffer their levels (Bahar Halpern et al., 2015; Müller et al., 2024; Steinbrecht et al., 2024). A recent manuscript described the accumulation of *NITRATE REDUCTASE 1* (*NIA1*) mRNA in response to nitrate at the site of transcription in the nucleus of Arabidopsis root cells (Fonseca et al., 2024). Importantly, this increased nuclear retention is associated with higher *NIA1* mRNA stability compared to cytoplasmic mRNA (Fonseca et al., 2024). This mechanism may also be important for *DOG1* mRNA levels in the *uba2a* mutant. Finally, nuclear localization of UBA2A disagrees with the observed increased stability of *DOG1* mRNA in the cytoplasmic fraction. One possible explanation could be the leakage of highly accumulated *DOG1* mRNA from *uba2a‐1* nuclei to the cytoplasm. Alternatively, we speculate that some post‐transcriptional modifications acquired during prolonged retention at the site of transcription, for example, extended poly(A) tails (Jia et al., 2022), may help to protect *DOG1* mRNA after export to the cytoplasm.

### UBA2A in seed biology

Here we report, the first to our knowledge, a factor that controls *DOG1* mRNA stability. The human homolog of Arabidopsis UBA2A, hnRNPAB, has been shown to block influenza RNA virus export from the nucleus (Wang et al., 2021, 2023). Our data show that also in plants, UBA2A is involved in subcellular mRNA localization control. UBA2A protein belongs to a conserved gene family that in Arabidopsis has two members: UBA2A and UBA2B. We show that despite the sequence homology, the *UBA2A* gene is not redundant with *UBA2B* in seed dormancy control. Phenotypic analysis of mutants in *UBA2A*, *UBA2B* and their double mutant combinations did not reveal obvious developmental defects apart from stronger seed dormancy. In agreement, genome‐wide transcriptome analysis of dry seeds showed very limited changes in gene expression, suggesting that the *UBA2A* gene is especially important for seed dormancy control.

Desiccation at the final stages of seed maturation has profound effects on gene expression regulation (Angelovici et al., 2010). Mutants of many general factors implicated in transcription and mRNA maturation show dormancy‐related phenotypes with only minor defects observed during other plant life stages under standard growing conditions (Grasser et al., 2009; Liu et al., 2011; Michl‐Holzinger et al., 2019; Layat et al., 2021). This may reflect the fact that mature seed chromatin is compacted due to water loss, and as a result, transcription during late seed maturation may be highly sensitive to Pol II‐related defects (Van Zanten et al., 2011; Layat et al., 2021). Moreover, maturing seeds are known to contain large amounts of stored mRNA (Sajeev et al., 2019), suggesting that regulation of mRNA metabolism is important at this developmental stage. Based on these results and our observations, we suggest that regulation at the transcriptional level may become less important during late seed maturation. We believe that post‐transcriptional effects, which are not so clear in other developmental stages, may become more evident at this exceptional stage of plant life.

Consistently, *UBA2A* knockout and overexpression lines have been reported to show no phenotypes, including germination of non‐dormant seeds (Lambermon et al., 2002). However, it is possible that under stress conditions, the *UBA2A* gene mutation could affect some post‐germination processes, as its human homolog hnRNPAB has been shown to regulate a specific set of processes, including influenza virus replication and cancer cells, malignancy (Wang et al., 2021, 2023). Given the evolutionary conservation of UBA2A in higher eukaryotes, we cannot exclude that in Arabidopsis, UBA2A could be involved in multiple stress‐related responses, especially in cases when transcriptional regulation is insufficient.

## MATERIALS AND METHODS

### Plant materials and growth conditions

The *uba2a‐1* (SALK_053281C N685951), *uba2a‐3* (SALK_045527 N545507), *uba2b‐1* (SAIL_788_B09C N835225), and *uba2b‐2* (SALK_013133 N513133) were obtained from Nottingham Arabidopsis Stock Centre (NASC). *dog1‐3* and *dog1‐5* were previously described (Fedak et al., 2016). All mutants used are in the *A. thaliana* Columbia‐0 (Col‐0) background. Double mutant plants (*uba2a‐1 uba2b‐2*, *uba2a‐3 uba2b‐2*, *uba2a‐1 dog1‐3*, *uba2a‐1 dog1‐5*, *uba2a‐3 dog1‐3*, *uba2a‐3 dog1‐5*) were generated by plant crossing. Plants were grown in an air‐conditioned greenhouse under a long‐day (LD) photoperiod (16 h of light at 22°C and 8 h of darkness at 18°C). Seeds were harvested and stored at room temperature for the specified periods.

The *uba2a‐1 35S::UBA2A‐GFP* (*green fluorescent protein*) plants were generated by cloning a 3.4‐kb fragment encompassing the entire coding region of *UBA2A* and a 1.9‐kb downstream flanking sequence into the N‐terminal GFP‐tag pGWB606 vector with a 35*S* promoter using Gateway cloning. Transgenic plants were produced using the floral dip method (Clough and Bent, 1998).

### Seed dormancy analysis

Eighty to 400 seeds were sown onto blue paper (Anchor) soaked in water and placed in a growing chamber at 22°C with a LD photoperiod. Germination was assessed daily for 1 week (the primary seed dormancy test) or 10 d (the secondary seed dormancy test). Seeds showing radicle protrusion were scored as germinated. For primary dormancy analysis, freshly harvested seeds were collected immediately after desiccation.

For secondary seed dormancy analysis, seeds were after‐ripened and tested for full loss of primary dormancy. Secondary dormancy induction was performed on water‐soaked blue paper by incubation at 30°C in darkness for 3 or 7 d before transfer to 22°C for germination analysis.

### RNA extraction and RT‐qPCR analysis

Total RNA from seeds was isolated using a phenol:chloroform extraction protocol (Cyrek et al., 2016; Kowalczyk et al., 2017). Four biological replicates were processed. After grinding 20–30 mg of seeds, samples were resuspended in 500 μL of homogenization buffer (100 mmol/L Tris pH 8.5, 5 mM ethylenediaminetetraacetic acid (EDTA), 100 mmol/L NaCl, 0.5% sodium dodecyl sulfate (SDS), 1% beta‐mercaptoethanol). Samples were centrifuged for 5 min at RT at 8,000 *g*. The supernatant was transferred to the new tubes. Then 250 μL of chloroform was added, and samples were shaken for 15 min. Next, 250 μL of phenol was added and shaking was repeated, followed by centrifugation for 10 min at 14,000 *g*. The upper phase was transferred to new tubes, and an equal volume of phenol:chloroform:isoamyl alcohol was added, shaken and centrifuged. The upper phase was mixed with 10% of the volume of 3 mol/L sodium acetate (pH 5.2) and an equal volume of isopropanol. After centrifugation for 15 min at 20,000 *g* at 4°C, the pellet was washed with 70% EtOH and resuspended in water. Subsequently, the TURBO DNA‐free kit (ThermoFisher) was used to digest DNA in the samples following the protocol provided by the manufacturer. To confirm that the DNA was successfully removed, PCR was performed using the primers for the PP2A gene (AT1G13320). Complementary DNA (cDNA) was synthesized from 1.5 μg of RNA using a SuperScript III RT kit (ThermoFisher) with oligo(dT) and random primers mixture. After 10‐fold dilution of cDNA, qPCR reaction was performed with LightCycler 480 SYBR Green I Master mix on the Roche LightCycler 480 instrument. Housekeeping gene *UBC21 (AT5G25760)* was used as a reference unless indicated otherwise. All primers are described in the Table S1. The relative transcript level for each gene was determined by the 2^−ΔΔCt^ method.

### Preparation of total protein extracts and western blot

Arabidopsis seeds were ground in liquid nitrogen and resuspended in 250 μL of extraction buffer (20 mmol/L Tris‐HCl pH 7.5, 2 mmol/L EDTA, 2 mM ethyleneglycoltetraacetic acid (EGTA), 50 mmol/L *β*‐glycerophosphate, 10 mmol/L dithiothreitol (DTT), 1 mmol/L phenylmethylsulfonyl fluoride, 1X cOmplete™ Protease Inhibitor Cocktail Roche, Switzerland). After short incubation, the extracts were centrifuged at 14,000 *g* for 30 min at 4°C, and the supernatant was transferred to new tubes. Thirty‐five micrograms of proteins (Bradford, 1976) from total protein extracts were separated on 10% SDS–polyacrylamide gels and transferred to a nitrocellulose membrane (Amersham, United Kingdom) using Trans‐Blot Turbo Transfer System 25 V 1 A 1 h. The membrane was blocked o/n at 4°C in TBST buffer (10 mmol/L Tris, pH 7.5, 100 mmol/L NaCl, and 0.1% Tween 20) containing 5% non‐fat milk, and then incubated o/n at 4°C in the same buffer with 1:2,000 diluted anti DOG1 antibodies, available from Agrisera (AS15 3032). After washing in TBST buffer, the membrane was incubated with 1:20,000 diluted alkaline phosphatase‐conjugated secondary antibody for 1 h at room temperature. After washing Pierce™ ECL Western Blotting Substrate (Thermo Scientific, USA) was added. The results were visualized by X‐ray film (Kodak, USA).

### RNA sequencing

RNA sequencing was done and analyzed using the 3′ RNA‐seq method as described in. Four biological replicates were processed. Briefly, RNA was isolated from dry after‐ripened seeds using a phenol:chloroform protocol and after DNase treatment, 500 ng of total RNA was used in reverse transcription with unique molecular markers‐containing oligo(dT) primers (Table S1) and SuperScript III kit (Thermo Fisher, USA). Libraries were sequenced on the Illumina NextSeq. 500 using pair‐end mode.

### Nuclei fractionation and chromatin RNA extraction

Chromatin RNA extraction was done using the method described in (Montez et al., 2023). Three biological replicates of dry seeds and six biological replicates of siliques collected during development were processed. Briefly, 100 mg of tissue were ground in liquid nitrogen and resuspended in 20 mL of cold Honda Buffer (20 mmol/L HEPES‐KOH pH 7.4, 0.44 mol/L sucrose, 1.25% Ficoll, 2.5% Dextran T40, 10 mmol/L MgCl_2_, 5 mmol/L DTT, 0.5% Triton X‐100, 10 mmol/L *β*‐mercaptoethanol, 1 mmol/L phenylmethylsulfonyl fluoride (PMSF), 1× Complete protease inhibitors (Roche), and 5 U murine RNase inhibitors). After rotation at 4°C for 10 min and filtration through Miracloth, samples were centrifuged at 2,000 g for 15 min at 4°C. The nuclei pellet was washed twice with 5 mL of Honda buffer. The pellet was resuspended in 600 μL of Honda buffer and purified on a 40%–75% PercoII density gradient by centrifugation at 10,000 *g* for 30 min at 4°C. Purified nuclei were collected from the Percoll interface and washed with Honda buffer and resuspended in 500 μL of chilled glycerol buffer (20 mmol/L Tris‐HCl pH 8.0, 75 mmol/L NaCl, 0.5 mmol/L EDTA, 0.85 mmol/L DTT, 50% glycerol, 1% Empigen, 10 mmol/L *β*‐mercaptoethanol, 0.125 mmol/L PMSF, 1 tablet/250 mL complete protease inhibitor, and 5 U murine RNase inhibitors). Nuclei were then overlaid on urea lysis buffer (10 mmol/L HEPES‐KOH pH 7.4, 7.5 mmol/L MgCl_2_, 0.2 mmol/L EDTA, 300 mmol/L NaCl, 1 mol/L urea, 1% NP‐40, 10 mmol/L *β*‐mercaptoethanol, 0.5 mmol/L PMSF, 1× Complete protease inhibitors (Roche), and 5 U murine RNase inhibitors), vortexed for 2 s, and incubated on ice for 30 min, followed by centrifugation at 20,000 *g* for 2 min at 4°C. The chromatin pellet was washed twice with 600 μL of urea lysis buffer for 30 min on a rotator in the cold. The chromatin pellet was used for RNA extraction and DNase treatment as described above. The chromatin‐attached RNA quality was tested by agarose gel electrophoresis and quantified with a Nanodrop 2000 spectrophotometer. The DNA contamination was evaluated by running PCR with the *PP2A* gene primers (AT1G13320), and afterwards, cDNA synthesis was performed using a SuperScript III kit (Thermo Fisher, USA).

### Pol II ChIP

Pol II ChIP was done as described with the following modifications (Kowalczyk et al., 2017). Four biological replicates were processed. One hundred milligrams of tissue (dry seeds or siliques collected during development) were ground in liquid nitrogen and accurately resuspended in 20 mL of crosslink buffer (10 mmol/L HEPES‐KOH pH 7.4, 50 mmol/L NaCl, 100 mmol/L sucrose, 1% formaldehyde). After 10 min incubation on a rotator at 4°C crosslink was rapidly quenched by adding 1.25 mL of 2 mol/L glycine. After 5 min incubation, samples were centrifuged at 4°C and 2,000 *g* for 15 min. The pellets were resuspended in 20 mL of cold Honda Buffer and filtered through Miracloth. Subsequently, samples were centrifuged for 15 min at 2,000 *g* at 4°C. The pellet was resuspended in ChIP lysis buffer (50 mmol/L Tris HCl pH 8; 10 mmol/L EDTA, 1% SDS, 1× complete protease inhibitors (Roche)) and sonicated using Bioruptor (Diagenode). After quality checking of sonicated chromatin, samples were diluted with ChIP dilution buffer (1.1% Triton X‐100; 1 mmol/L EDTA; 16.7 mmol/L HEPES‐KOH pH 7.4, 167 mmol/L NaCl; 1 mmol/L DTT, 1 mmol/L PMSF; 1× Complete protease inhibitors (Roche)) and incubated with RNA polymerase II subunit B1 antibodies (Agrisera) and Dynabeads Protein G beads (Thermo Fisher, USA) overnight on a rotator at 4°C. After washing with low salt (150 mmol/L NaCl; 1 mmol/L EDTA; 10 mmol/L HEPES‐KOH pH 7.4, 0.1% Triton X‐100) and high salt (500 mmol/L NaCl; 1 mmol/L EDTA; 10 mmol/L HEPES‐KOH pH 7.4, 0.1% Triton X‐100) buffers, DNA was eluted and reverse‐crosslinked by incubation in 95°C with 10% Chelex 100 solution for 15 min. After Proteinase K treatment and centrifugation, the clean DNA was used in a qPCR reaction with appropriate primers as listed in Table S1.

### Single‐molecule RNA FISH

The smFISH was done as described in (Montez et al., 2023) using a manually dissected radicle from dry embryos. The Stellaris probes target the full *shDOG1* sequence, including intron 1 and are labeled with Quasar670 fluorophore (Biosearch Technologies, United Kingdom). The embryos were fixed, permeabilized and hybridized with the probes. The smFISH signals were imaged using a widefield fluorescence microscope Olympus IX81 (Olympus, Japan) and a Hamamatsu Orca‐R2 (C10600) charge‐coupled device camera. The xCellence software (Olympus, Japan) was used for image acquisition. Cells were manually segmented using Napari (Sofroniew et al., 2022) and the PartSeq plugin, which allows foci identification and classification based on subcellular location (Bokota et al., 2021). Dot numbers and intensity were analyzed using R scripts as described (Montez et al., 2023).

### Flavopiridol RNA stability assay

Seven‐d‐old seedlings were sprayed with flavopiridol (20 μmol/L flavopiridol, 0.02% Silwet‐77). Four biological replicates were processed. One gram of material was ground with liquid nitrogen and resuspended in 12 mL of Honda buffer. Then, 500 μL of the extract was proceeded directly to RNA extraction as the total cell fraction. The rest was filtered through a Miracloth and centrifuged to sediment the nuclei (as described in the Nuclei fractionation and chromatin RNA extraction section). From the supernatant, 500 μL was collected as the cytoplasm fraction. The nuclei pellet was washed twice with 2 mL of Honda buffer and lyzed (as described in the Nuclei fractionation and chromatin RNA extraction section). After 30 min of ice incubation, the samples were centrifuged, and the chromatin pellet was resuspended in 500 μL of RNA homogenization buffer. After chloroform followed by phenol extraction (see RNA extraction and RT‐qPCR analysis), RNA was precipitated and resuspended in 500 μL of TriSure (Applichem, Germany). After the TriSure purification (according to the manufacturer's protocol), RNA was digested with DNase Turbo (Thermo Fisher, USA) and recovered by phenol:chloroform:isoamyl (24:24:1) extraction followed by isopropanol precipitation and washed in 70% ethanol. The cDNA primed with oligo‐dT was analyzed with qPCR using transcript‐specific primers (see Table S1), as described above. For each primer pair, a standard curve was created using serial dilutions of the chromatin fraction. Absolute values were calculated based on standard curve parameters. The half‐lives were determined using the formula −1/*a*, where “*a*” represents the coefficient derived from fitting a linear model to the Log_2_ of transcript levels over time. The statistical significance was calculated using the *lsmeans* R package (Lenth, 2016).

## CONFLICTS OF INTEREST

The authors declare no competing interests.

## AUTHOR CONTRIBUTIONS

C.W. performed the majority of the experiments, L.B. did the RNA stability assay, and Se.S. carried out fractionation experiments. M.K. performed and analyzed RNA‐seq and Pol II ChIP, V.H.M. analyzed *UBA2A* complementation lines, M.J.O. and A.K. performed the western blot, and C.W. and S.S. wrote the manuscript and analyzed the data. All authors read and approved the contents of this paper. The authors like to announce that in their opinion L.B., Se.S., and M.K. contributed equally to this work.

## Supporting information

Additional Supporting Information may be found online in the supporting information tab for this article: http://onlinelibrary.wiley.com/doi/10.1111/jipb.70056/suppinfo

**Figure S1.** UBA2A negatively regulates primary and secondary seed dormancy
**Figure S2.** *uba2a*, *uba2b* or *uba2 uba2b* mutants do not show obvious developmental phenotypes
**Figure S3.** UBA2A requires the functional *DOG1* gene for primary seed dormancy control
**Figure S4.** UBA2A does not control *DOG1* transcription
**Figure S5.** *uba2a* shows increased chromatin retention of *DOG1* transcripts

**Table S1**. Primers used in this study

**Table S2**. Additional information for the RNA sequencing

## Data Availability

Data supporting this work are available in this article and the Supporting Information. 3′ RNA‐seq data were submitted to the Gene Expression Omnibus repository (GSE245457).
