# Supplementary material for: UBA2A regulates seed dormancy and the stability of chromatin‐retained DOG1 messenger RNA
Source: J Integr Plant Biol. 2025 Oct 23;67(12):3109–22. doi: 10.1111/jipb.70056 (PMC12678680; doi:10.1111/jipb.70056)
Supplement: Supplementary file 1 — Figure S1. UBA2A negatively regulates primary and secondary seed dormancy Figure S2. uba2a, uba2b or uba2 uba2b mutants do not show obvious developmental phenotypes Figure S3. UBA2A requires the functional DOG1 gene for primary seed dormancy control Figure S4. UBA2A does not control DOG1 transcription Figure S5. uba2a shows increased chromatin retention of DOG1 transcripts [file JIPB-67-3109-s002.docx]

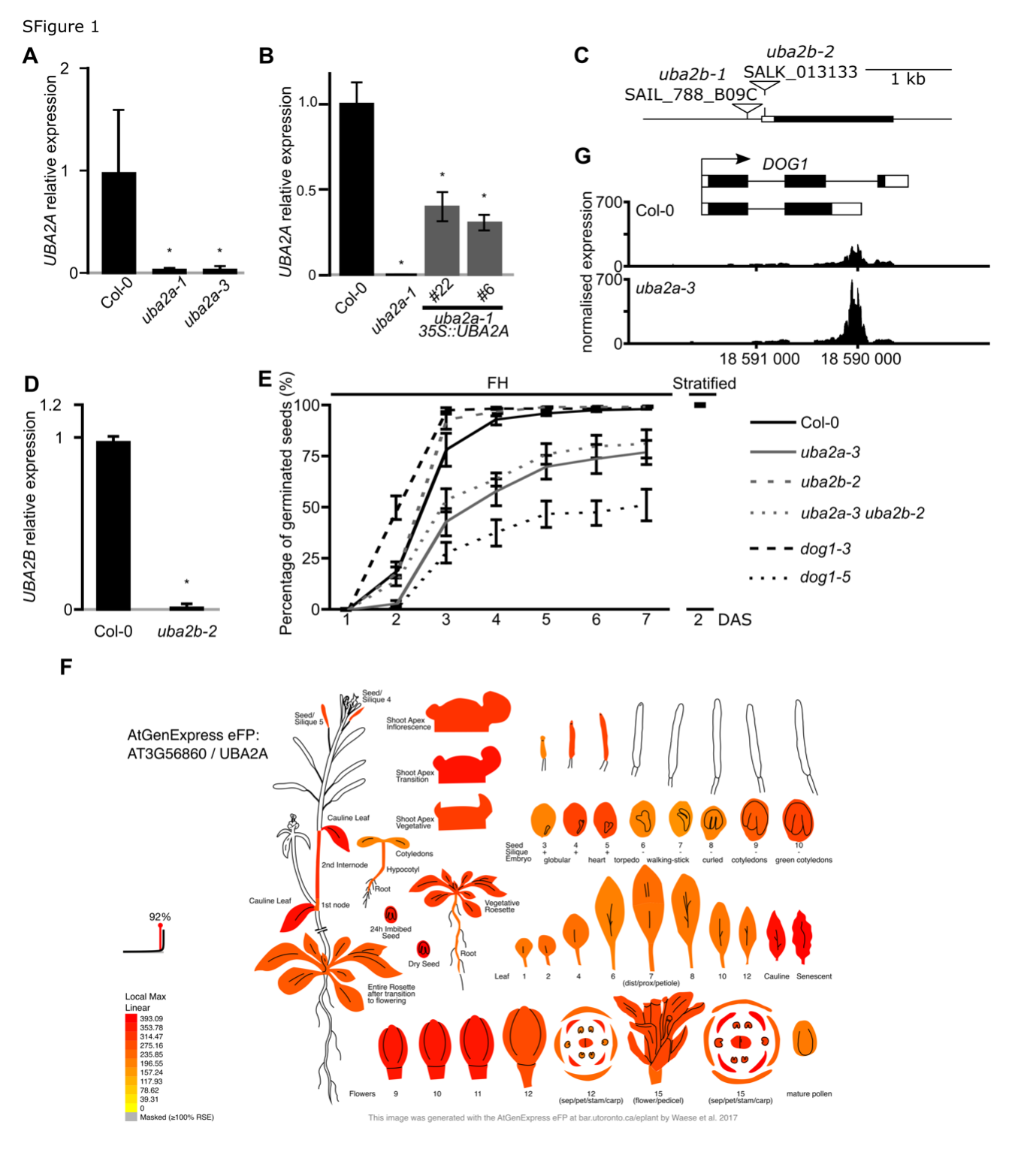


**Figure S1** UBA2A negatively regulates primary and secondary seed dormancy

**(A)** RT-qPCR was used to analyse the expression levels of UBA2A in *uba2a* mutants. **(B)** RT-qPCR was used to analyse the expression of *UBA2A* in complementation experiment. **(C)** UBA2B gene structure with positions of T-DNA insertion of mutants used in this work. A black rectangle corresponds to an exon, white rectangles correspond to UTRs. **(D)** RT-qPCR was used to analyse the expression levels of UBA2B. **(E)** *uba2b* mutant shows no major change in primary seed dormancy and *uba2a-3 uba2b-2* double mutant shows similar dormancy defects as the *uba2a-3* single mutant. Freshly harvested (FH) seeds were sown and scored for germination for 7 days (1-7 DAS). A stratification control is shown on the right side of the graph for the same lot of seeds.**(F)** AtGenExpress eFP browser data for *UBA2A* expression. **(G)** 3’RNA-seq reads for *DOG1* gene in Col-0 and *uba2a-3*. The upper panel shows the *DOG1* gene structure with exons shown as black rectangles, introns as lines and UTRs as white rectangles. The lower panel shows the normalised number of reads corresponding to positions along the *DOG1* gene and axis labelling shows chromosome position.Panels A,B,D, and E show the mean from 4 biological replicates. Error bars denote standard deviations.


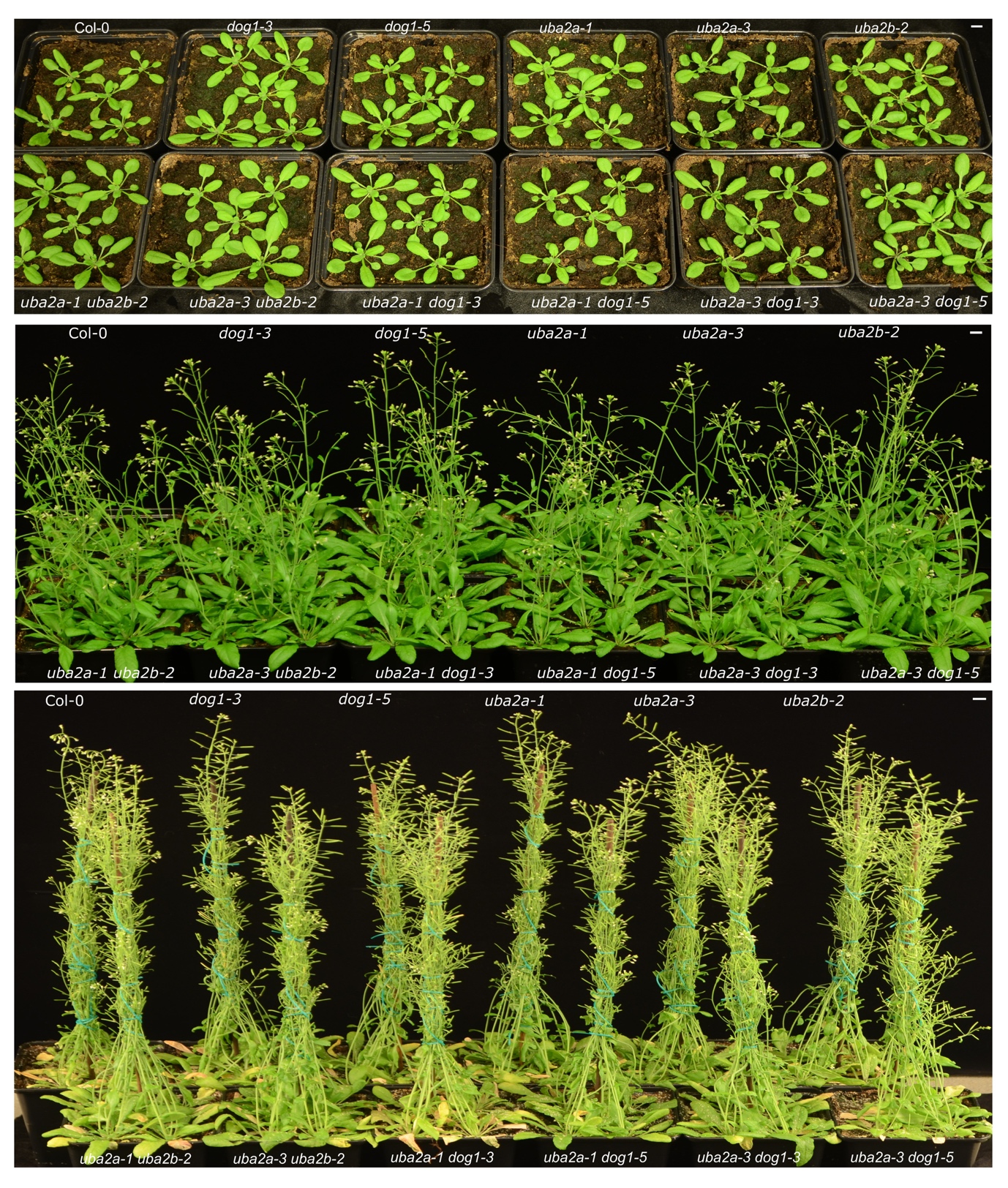


**Figure S2** *uba2a*, *uba2b* or *uba2 uba2b* mutants do not show obvious developmental phenotypes.

Pictures were taken at the seedling stage (top panel), flowering stage (middle panel) and seed maturation stage (bottom panel). Scale bars, 1 cm.


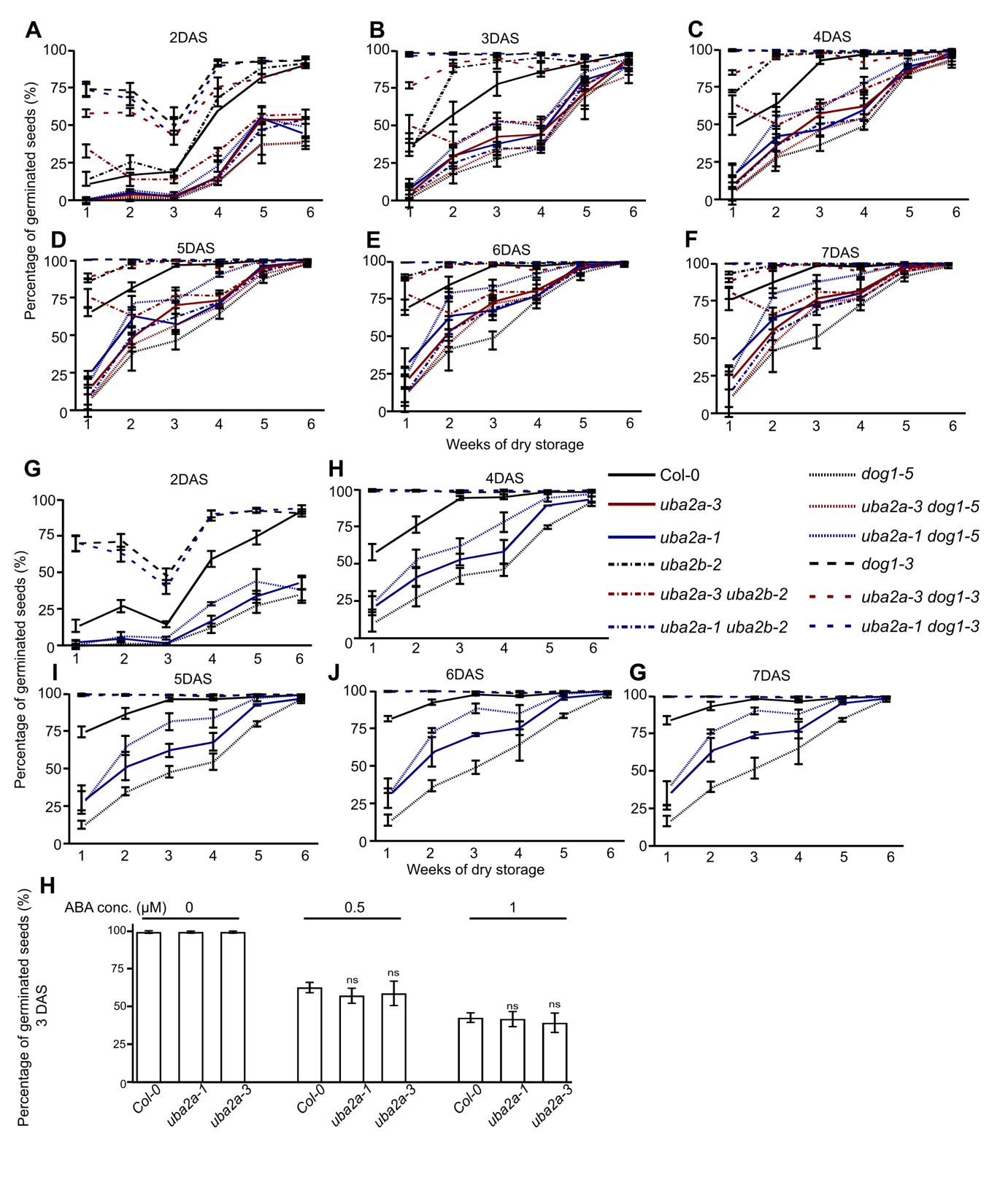


**Figure S3**  UBA2A requires the functional *DOG1* gene for primary seed dormancy control.

Seeds were stored in dry conditions for the indicated time and scored for germination at the **(A)** 2, **(B)** 3, **(C)** 4, **(D)** 5, **(E)** 6 and **(F)** 7 DAS for *uba2a* and *uba2b* and double mutants indicated. Seeds were stored in dry conditions for the indicated time and scored for germination at the **(G)** 2, **(H)** 4, **(I)** 5, **(J)** 6 DAS for *uba2a* and *uba2b* and double mutants indicated. Figure 3C, D contains a selection of data shown in full here. All graphs show the mean germination percentage calculated from 4 biological repeats. Error bars denote standard deviations. **(H)** Percentage germinated seeds for after-ripen seeds stratified for 2 days and germinated for 3 days at the ABA concentrations indicated.


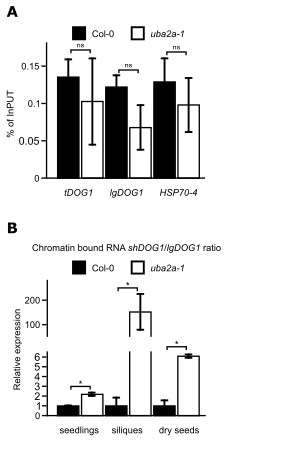


**Figure S4** UBA2A does not control *DOG1* transcription

**(A)** Pol II ChIP in developing seeds. Data represent percent of input normalised to Col-0 (average from 3 biological replicates) and error bars denote standard deviations. Primers to ACT7 (AT5G09810) gene promoter were used as a reference. **(B)** RT-qPCR analysis on chromatin-attached RNA was used to analyse shDOG1/lgDOG1 ratio in seedlings, siliques and seeds. Data represent mean from 3 biological replicates and error bars denote standard deviations.


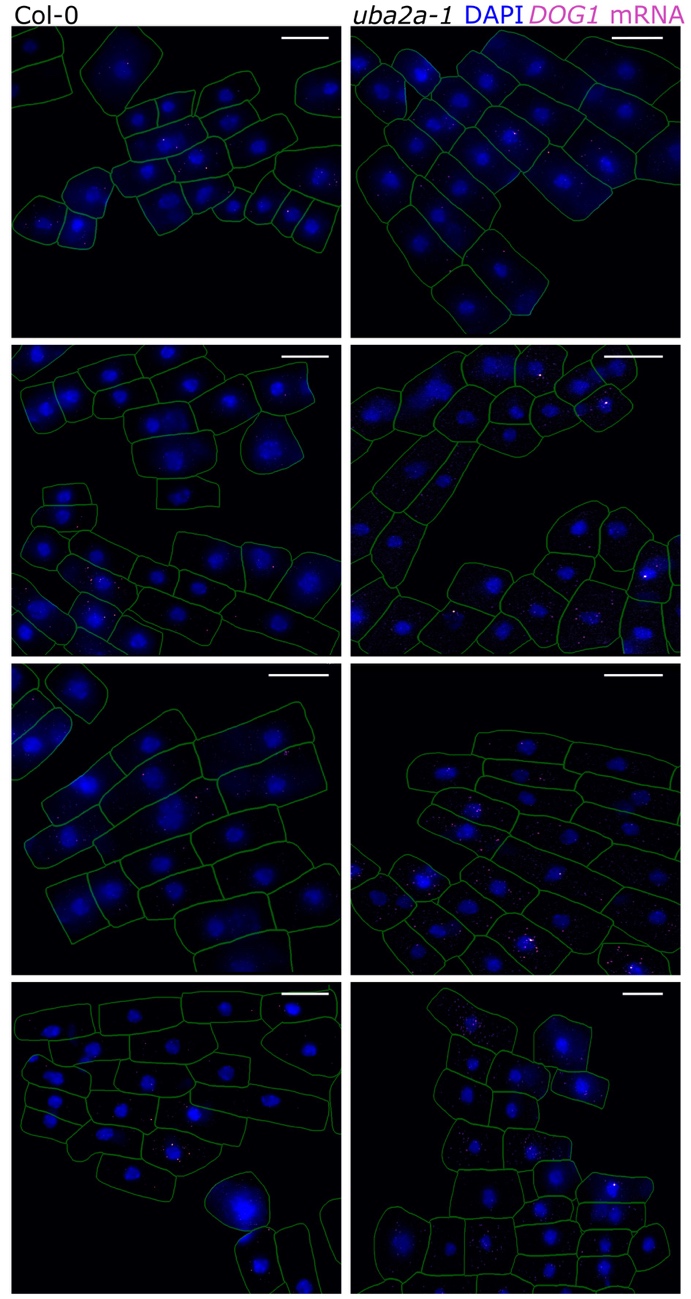


**Figure S5** *uba2a* shows increased chromatin retention of *DOG1* transcripts.

Single-molecule RNA fluorescence in situ hybridization (smFISH) imaging of *DOG1* RNA in seed cells. A collection of representative pictures of the root tip for Col-0 and *uba2a-1* mutant seeds (left and right panels respectively). The cell contour is shown as green line, DAPI staining in blue and *DOG1* signal in magenta-to-white colour scale. Scale bars, 10 µM.
